# Supplementary figures and images for: DrugDiff: small molecule diffusion model with flexible guidance towards molecular properties
Source: J Cheminform. 2025 Feb 25;17:23. doi: 10.1186/s13321-025-00965-x (PMC11854002; doi:10.1186/s13321-025-00965-x)

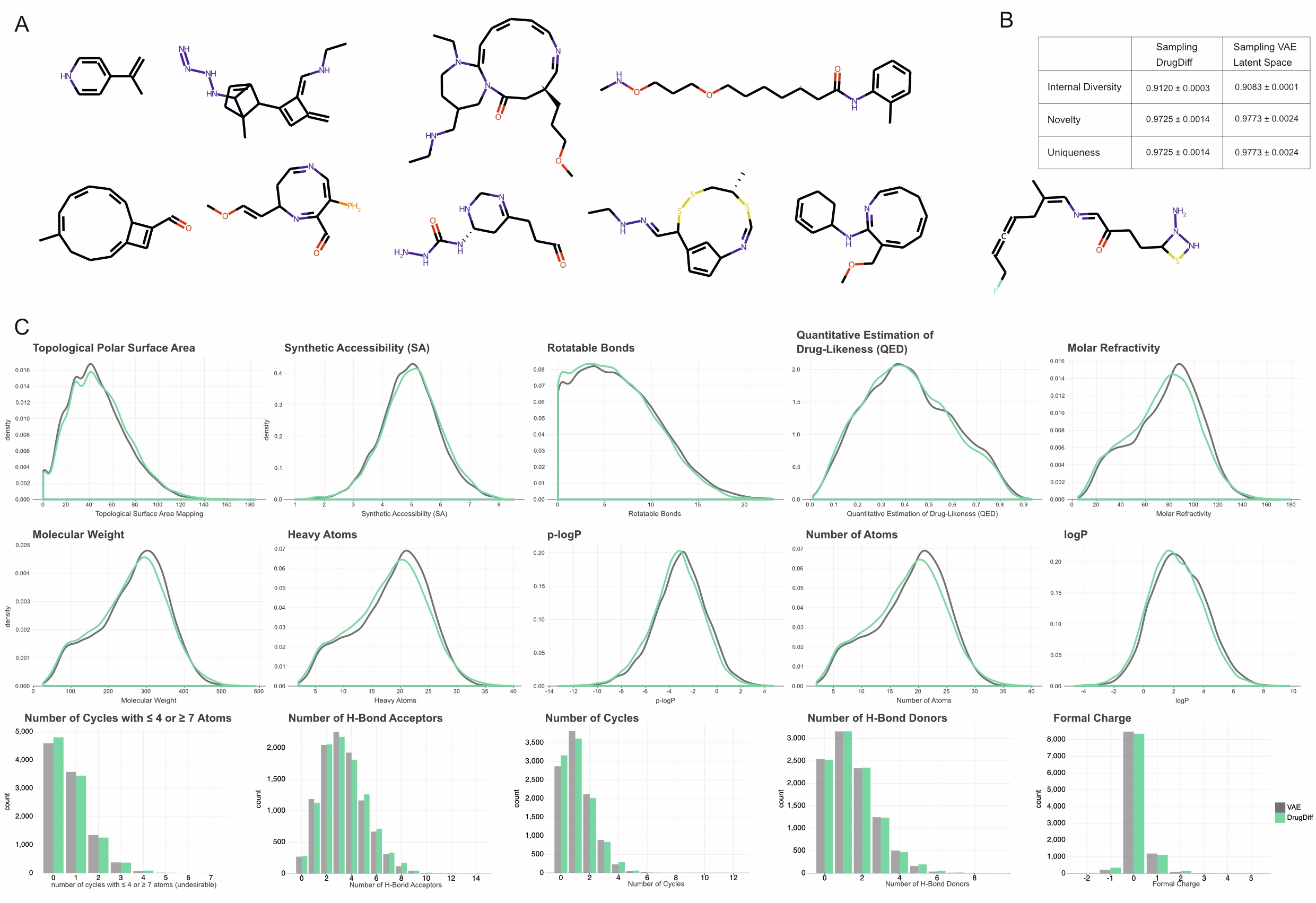

Supplement: Supplementary file 1 — Suplplemenaty material 1. [file 13321_2025_965_MOESM1_ESM.jpg]

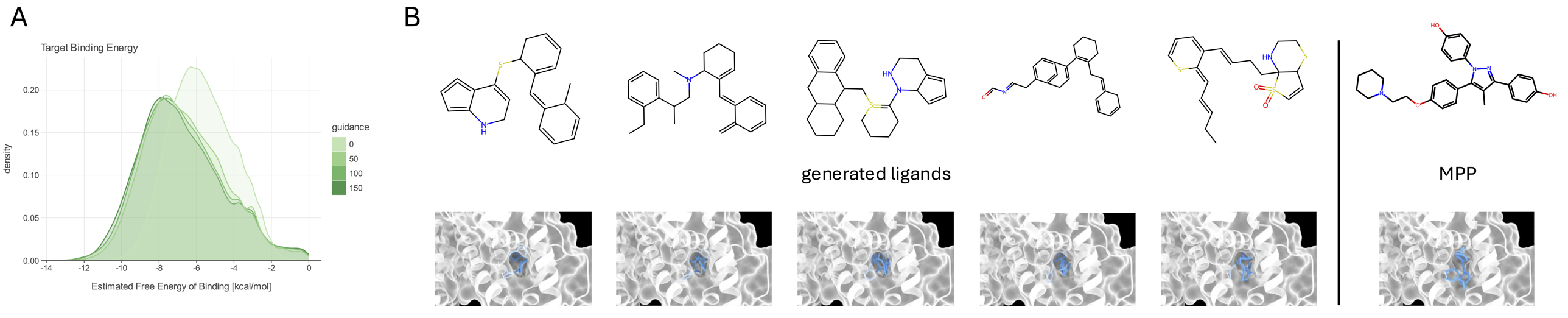

Supplement: Supplementary file 2 — Suplplemenaty material 2. [file 13321_2025_965_MOESM2_ESM.png]
