# Supplementary material for: DrugDiff: small molecule diffusion model with flexible guidance towards molecular properties
Source: J Cheminform. 2025 Feb 25;17:23. doi: 10.1186/s13321-025-00965-x (PMC11854002; doi:10.1186/s13321-025-00965-x)
Supplement: Supplementary file 3 — Suplplemenaty material 3. [file 13321_2025_965_MOESM3_ESM.docx]

# Supplementary Information

|  | Validity | Uniqueness | Novelty | KL div | FCD |
| --- | --- | --- | --- | --- | --- |
| VAE | 0.9997±0.0001 | 0.9857±0.0014 | 0.9967±0.0003 | 0.6542±0.0056 | 0.0037±0.0000 |
| DrugDiff | 0.9998±0.0002 | 0.9869±0.0021 | 0.9970±0.0003 | 0.6667±0.0007 | 0.0036±0.0000 |

**Supplementary Table 1: GuacaMol distribution learning benchmark.** VAE was re-trained on the GuacaMol benchmarking set, *DrugDiff* was then re-trained on the VAE latent space. The similarity of the benchmark results emphasise that *DrugDiff* is capable of fully learning the VAE’s latent space. KL: Kullback-Leibler Divergence. FCD: Fréchet ChemNet Distance.

| Model | QED (↑) | SA (↓) |
| --- | --- | --- |
| GCPN | 0.65±0.15 | 4.53±0.86 |
| JTVAE | 0.64±0.14 | 4.69±0.76 |
| MolGPT | 0.67±0.16 | 3.98±0.99 |
| MolGAN | 0.33±0.00 | 5.23±0.00 |
| GraphDF | 0.42±0.13 | 4.80±0.91 |
| LSTM | 0.73±0.14 | **2.47**±0.88 |
| Taiga | **0.75**±0.11 | 2.89±0.92 |
| DrugDiff | 0.53±0.15 | 4.23±0.72 |

**Supplementary Table 2: Optimisation Benchmark.** Shown are the mean values and standard deviation for two optimisation tasks: drug-likeness (QED) and synthetic accessibility (SA). The best performing value is highlighted in bold. Scores were computed based on 25,000 molecules generated per experiment. This is in accordance with the extensive comparison provided by [[32]](https://sciwheel.com/work/citation?ids=15742128&pre=&suf=&sa=0&dbf=0) from which we have retrieved the reference values for all models other than *DrugDiff*.


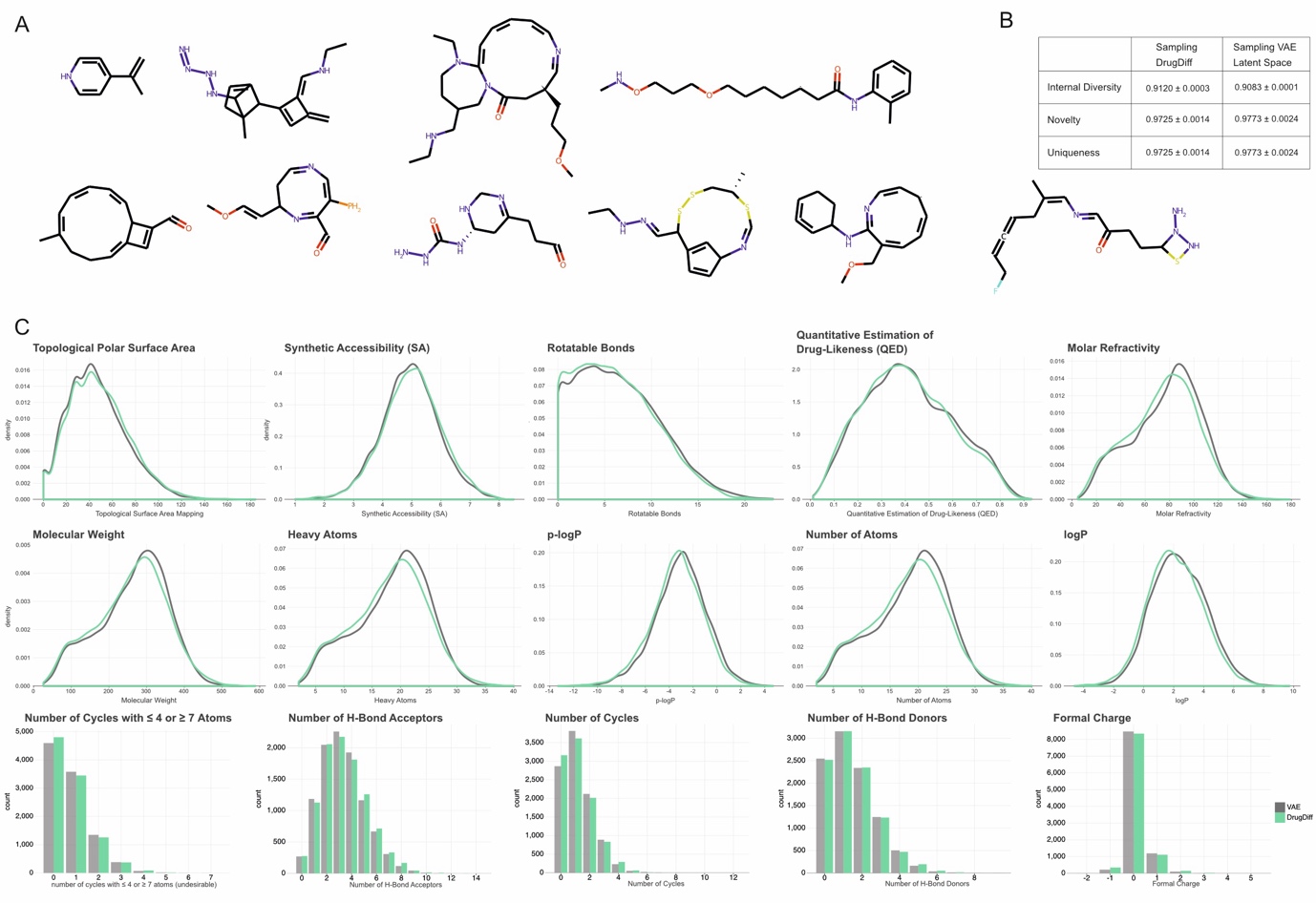


**Supplementary Figure 1: Unguided Generation.** A) Randomly chosen subset of molecules generated by *DrugDiff* without guidance; B) Internal diversity, novelty and uniqueness of molecules generated with *DrugDiff* vs. directly sampled from the VAE latent space. Results are shown as mean and standard deviation from 3 generated sets of 10,000 molecules each. All metrics are on a scale from 0 to 1 with 1 being the optimum; C) Distribution of different molecular properties in 10,000 molecules generated with *DrugDiff* (green) vs. directly sampled from the VAE latent space (grey).


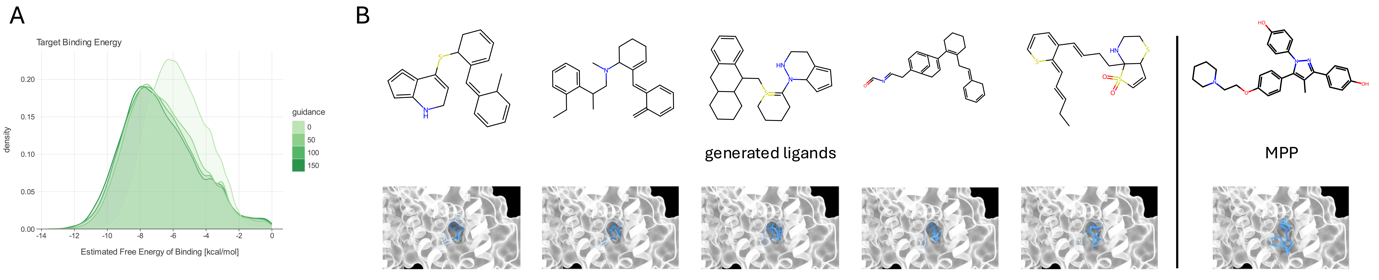


**Supplementary Figure 2: ESR1 Predictor Training and Generated Ligands.** A) Distribution of estimated free binding energies computed with AutoDock-GPU for 10,000 molecules generated by *DrugDiff* under different guidance strengths (0, 50, 100, 150) towards lower energies (analogous to higher affinity). B) Top 5 generated ligands based on the free biding energies as well as Methylpiperidinopyrazole (MPP), a known antagonist for *ESR1*. Below, the binding sites of the best found docking poses are shown.
